# Sustainable Patient Education in Urology: Evaluating Quick Response (QR) Code-Based Information Delivery

**DOI:** 10.7759/cureus.98645

**Published:** 2025-12-07

**Authors:** Sabin Yadav, Hrishi Joshi, Krishna Narahari, Mudassir Wani

**Affiliations:** 1 Urology, University Hospital of Wales, Cardiff, GBR

**Keywords:** digital leaflets, patient information leaflet, qr code, quality improvement tool, sustainable practices

## Abstract

Background

Clear communication and effective dissemination of information remain the mainstay of any clinical appointment. This allows the clinician and the patient to formulate a management plan with informed consent from the patient. The British Association of Urological Surgeons (BAUS) provides validated patient information leaflets (PILs) that aid in this discussion. Our study evaluated the practicality and patient satisfaction of using quick response (QR) codes for the dissemination of PIL as a means to reduce our carbon footprint.

Method

We performed a prospective short quality improvement study in two phases. For the first phase, we collected data from consecutive patients over a period of one week to be able to include all types of clinics, and we focused on the feasibility of using QR codes for PIL in our patient cohort. For the second phase, we collected data over a month and included patients who were provided with a QR code for the PIL during previous consultations. During this phase, we focused on patient satisfaction after implementing the QR code for PIL.

Results

For the first phase, 77 patients were included, 91% (n=70) male with a median age of 76 years (range 20-90), 87% (n=67) were using a smartphone. 74% (n=57) were aware of QR codes, and 64% (n=49) were willing to use QR codes for PIL. For the second phase, 50 patients were included, 90% male with a median age of 54 years (range 31-70). 100% (n=50) of patients agreed QR codes were easy and convenient to use. 80% (n=40) preferred QR codes to paper PIL.

Conclusion

The use of QR code PILs is gaining acceptance, especially with the increasing use of smartphones. While printed copies should be available for those who prefer them, QR codes offer a sustainable and cost-effective alternative. Within the limitations of our study, patients showed a willingness to adopt QR codes and provided positive feedback, but given the small sample size and short study period, these findings should be interpreted cautiously. The transition to QR codes would support environmental goals, reduce expenses, and ensure privacy while effectively delivering validated information to patients.

## Introduction

Clear communication and effective dissemination of information are crucial for enhancing patients' understanding of their medical condition, which has been found to improve engagement and adherence to the treatment plan, as well as managing patient expectations, as explained by Jimmy and Jose [1]. However, patients often misunderstand the information provided to them, which leads to errors in management plans, as evidenced in a study by the National Academies of Sciences, Engineering, and Medicine, 2018 [2]. The task of promoting patient understanding of diagnostic, therapeutic, and surgical interventions is often complex due to potential barriers, such as the patient's health literacy and the healthcare provider's communication skills [3]. These barriers can result in information being forgotten or remembered incorrectly by the patient. To overcome this essential aspect of clinical management, evidence confirms that written information helps patients to understand and adhere to treatment [4].

The British Association of Urological Surgeons (BAUS) has developed validated patient information leaflets (PILs) for various urological conditions and procedures. These validated PILs are updated regularly and ensure consistency in providing updated information to all patients across the United Kingdom [5]. Lately, quick response (QR) codes have seen their utilisation in healthcare in various forms, which include track and trace for diseases like COVID, patient identification bracelets, and identifiers for samples (vacutainer, histopathology) [6]. BAUS PILs are also available in QR code format. QR codes have the potential to be used to disseminate validated PILs to patients without any added cost and decreasing paper consumption, i.e., reducing our carbon footprint.

This short prospective closed-loop quality improvement study aimed to evaluate the feasibility of QR codes for PIL in the first phase and the acceptance of use of QR codes for PIL in the second phase. We also reviewed the potential benefits and barriers to using QR codes for PIL.

## Materials and methods

We performed a short closed-loop prospective quality improvement study in the urology outpatient department at University Hospital Wales, Cardiff, UK. This study was registered with the hospital research and audit department (registration code: Urology/SE/2024-25/02). We used questionnaires for this study, which were not formally validated but reviewed internally by the clinical team to ensure clarity and relevance (see Appendix). The study was conducted in two phases.

First phase

It was conducted over one week (19-23 August 2024) and included all patients consecutively attending the urology outpatient clinics. We performed this study over a period of one week to be able to include all kinds of clinics at our centre. During this phase, data were collected after verbal consent. Participation was voluntary, and there were no exclusion criteria due to the nature of the study. We used a questionnaire with three subsections to collect information. The first section included patient demographics, i.e., age and sex. The second section included questions about the use of smartphones with internet access, awareness of QR codes, and willingness to use QR codes for validated patient information leaflets, both of which were filled in by the patients while waiting for their appointments, and the third section included details of the PIL provided, which was completed by the clinician post appointment. We printed the questionnaire on A4 paper; two questionnaires were printed on one sheet to minimise paper usage. After receiving positive responses from most of our patients, we started offering QR codes to patients for PIL.

Second phase

Patients attending our outpatient clinic over a one-month period (January 2025) who were provided with QR codes during the previous consultation were included in this study. Those who did not receive a QR code at their prior appointments were excluded from this study. Participation was voluntary, and data were collected with verbal consent. Patients completed this when waiting for their appointment. Information on patient demographics (including age and sex) was recorded, and a five-point Likert-scale questionnaire was used to assess patients' perceived ease of using QR codes, their perceived convenience compared with conventional paper PILs, and their preference between QR-code and paper PIL formats.

For this study, we defined "ease" as the patient's perceived simplicity of accessing and viewing the PIL using the QR code. "Convenience" referred to how practical and time-efficient patients found the QR-code format compared with paper leaflets. "Preference" reflected whether patients favoured QR-code PILs or traditional paper PILs as their primary source of information. These constructs were assessed using a five-point Likert-scale questionnaire, which provided a structured and consistent method for evaluating patient perceptions.

Ethical considerations

This study was conducted as a quality improvement study and did not require formal ethical approval. Participation was voluntary, and verbal informed consent was obtained from all participants before data collection. All patient data were anonymised to ensure confidentiality and privacy.

## Results

First phase

The first cycle of our study aimed to assess the awareness of QR codes amongst our patient cohort and the willingness of our patient cohort to switch to QR codes. Seventy-seven patients attended the outpatient clinic in a period of one week, and all were included in this study. As seen in Table 1, our cohort comprised mostly male patients (n=70, 91%) with a median age of 76 years (range: 20-90). The majority of this group were using a smartphone with internet access (n=67, 87%), 74% (n=57) were aware of QR codes, and 64% (n=49) were willing to use QR codes for PIL. As shown in Table 2, if we were to only consider patients with a smartphone and access to the internet, 83.5% (n=56) of patients were aware of QR codes, and 73% (49) were willing to use QR codes instead of paper for PIL. Forty-eight paper PIL were handed out to patients over the period of this week, with a total of 312 A4 papers.

**Table 1:** Demographics, use of smartphones with internet access, awareness of QR codes, and willingness to use QR codes for PIL. QR: quick response; PIL: patient information leaflet.

Variable	Category	N (%)
Age (years)	76 years (20-90)	
Gender	Male	70 (91%)
	Female	7 (9%)
Use of smartphone with internet access	Yes	67 (87%)
	No	10 (13%)
Awareness of QR codes	Yes	57 (74%)
	No	20 (26%)
Willingness to use QR codes	Yes	49 (64%)
	No	28 (36%)

**Table 2:** Subset analysis of patients with a smartphone and internet access. QR: quick response.

Variable	Category	N (%)
Use of smartphone with internet access	67	
Awareness of QR codes	Yes	56 (83.5%)
	No	11 (16.5%)
Willingness to use QR codes	Yes	49 (73%)
	No	18 (27%)

Second phase

**Figure 1:**
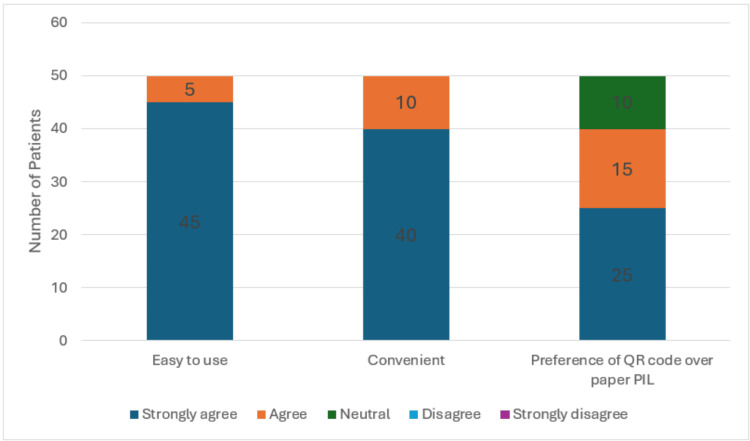
Ease of use, convenience, preference of QR code over paper PIL (second phase). QR: quick response; PIL: patient information leaflet.

The second cycle of our study was aimed at evaluating patient satisfaction with using QR codes instead of paper PIL. As shown in Table 3, a total of 50 patients were included in this cycle, with mostly male patients (n=45, 90%). The median age was 54 years (range: 31-70). As shown in Figure 1, three domains were examined: ease of use, convenience, and preference of QR code to paper PIL. 100% (n=50) of respondents agreed that QR codes were easy and convenient to use. Most patients in our cohort (n=40, 80%) preferred QR codes over paper PIL, whereas the remaining were neutral. No patients in this cohort had a strong preference for paper PIL.

**Table 3:** Demographics of the second phase.

Variable	Category	N (%)
Age (years)	54 years (31-70)	
Gender	Male	45 (90%)
	Female	5 (10%)

## Discussion

Effective communication with patients is vital for high-quality healthcare in all specialties, where decisions involve long-term management and complex procedures. Studies have shown that memory for medical information provided during a clinical consultation is often inaccurate and poor, particularly for diagnoses that have a long-term impact on a patient's life [4,7]. Patients tend to remember written information better compared to verbal information [4,8], and BAUS PIL's play a crucial role in improving patients' understanding, which supports informed decision-making and adherence to treatment plans.

In 1994, Denso Wave developed QR codes to track car components during manufacturing and distribution. Since then, the use of these pixelated squares has rapidly increased across various fields. It has also been widely incorporated into healthcare [9]. We wanted to switch to QR codes for PIL for urology practice in the UK. QR codes could direct users to a website containing the leaflets, which, in our case, was from the BAUS website. The website is regularly updated by the BAUS team, so QR codes can link to the updated PIL without the need to generate a new QR code every time the PIL is updated.

The first phase revealed that the majority of our patient cohort were aware of how to use QR codes and expressed willingness to switch to QR codes for PIL. However, we observed that elderly patients were less likely to be aware of or be able to use QR codes, which is in line with a recent study [10]. This reflects a broader challenge in health equity where age, socioeconomic status, and digital literacy may affect the uptake of technological interventions. Lyles et al. [11] highlighted that equitable digital transformation must ensure accessibility for all patients, including those less digitally confident. For this reason, retaining the option of printed PILs remains essential to avoid excluding vulnerable groups.

We also found 48 paper PIL were handed out to patients over the period of this week, with a total of 312 A4 papers, which can be used to project the use of 17,472 A4 sheets from the in-person clinic alone. Our centre also sent out PIL via post for virtual patients and patients seen during on-call, so the actual number of paper usage for PIL is larger than this projected value.

The second phase was to evaluate patient satisfaction with switching from paper PIL to QR codes. The majority of patients in this cohort found the QR code convenient and easy to use. The median age for this cohort was slightly lower compared to the first cycle, with a median age of 54 years. The findings favouring QR codes in this age group are also in line with other studies [12]. During the second cycle, we also received a lot of positive feedback for this initiative, but there were concerns raised regarding the need for the option of paper PIL, as some of our patients might be differently abled. We also received suggestions regarding the availability of leaflets in other languages to facilitate the needs of patients from other geographical areas (e.g., Welsh for Wales). This highlights an important consideration of digital health equity where differences in age, digital literacy, accessibility, and socioeconomic background may influence patients' ability to engage with technology-based health tools [13]. As Lyles et al. (2022) [11] emphasised, achieving digital transformation in healthcare requires ensuring equitable access so that digital innovations do not inadvertently widen existing health disparities.

In addition, patients shared that the use of QR codes was better for privacy. Unlike paper PILs, which required careful handling and could be misplaced at home, QR codes allowed them to store the same information securely on their smartphones, which were mostly password-protected, hence enhancing privacy and convenience.

Globally, healthcare contributes approximately 5% of total greenhouse emissions [14]. The carbon footprint of one A4 paper is estimated to be approximately 4.64 g CO2 [15], which would mean 81,075 g of CO2 or 0.081 tonnes of CO2 for our projected use of 17,472 A4 sheets in clinic over one year period, this would be equivalent to driving a mid-range car with two passengers for 6.5 hours or a one way flight in economy class from London to Paris [16]. By switching to QR codes for patient information leaflets, we can significantly reduce paper usage and make a simple yet meaningful contribution to environmental sustainability. This also aligns with wider environmental sustainability goals. The NHS has committed to a net-zero carbon footprint [17], and the utilisation of digital communication strategies, such as QR codes, makes a meaningful contribution to this target and is very easily reproducible. While QR codes would help to reducing our environmental impact, it is also essential to acknowledge that digital solutions are not entirely carbon neutral as the production and operation of devices, storage servers, and the energy required for data transfers also contribute to the carbon footprint.

In our NHS trust, we have started offering QR codes for all our patients, with an option to choose paper PIL if that remains the patient's preference. We have also started putting the QR codes on our letters to the patients for relevant procedures. We have provided the relevant QR codes to the secretarial team, and they are also able to create their own QR codes, as it is just a simple step with a right click on the browsing page of a web browser and selecting create a QR code.

While advocating for switching to QR codes instead of paper leaflets, we are aware that all our patients might not be comfortable with using QR codes for various reasons. We would therefore recommend offering patients an option for using QR codes and conventional PIL to avoid any form of discrimination.

Limitations

There are limitations to consider when interpreting the findings for this quality improvement study. The short study period and small sample size, particularly the differences in sample size between the two phases, might limit the generalisability of our findings. The study was conducted at a single centre, and the predominance of male participants may have skewed results and overpowered the representativeness of female perspectives. Additionally, the questionnaire was not formally validated, and statistical analysis was limited to descriptive reporting, which restricts the strength of conclusions. While all eligible patients were included in each phase, differences in digital literacy and familiarity with QR codes may have influenced patient responses and willingness to engage with the intervention. These factors should be considered when interpreting the results, and future studies with larger, more diverse populations and validated instruments are warranted.

## Conclusions

Our study, within its limitations, suggests that our patients are willing to use QR codes for PIL and found QR codes easy and convenient. Offering both QR-code and conventional paper PILs remains important to ensure equitable access to validated information. QR codes have the potential to support more sustainable information delivery. They may enhance privacy by reducing the risk of misplaced or visible personal health information, but further studies with larger, more diverse populations are needed to confirm these findings and fully assess their impact.

## Appendices

**Table 4:** First phase questionnaire.

Section 1 (Demographics)		
Clinic date:		
Age:		
Gender:		
Section 2 (Please circle your answers)		
1. Do you have a smartphone with Internet access?	Yes	No
2. Are you aware of QR codes? (Do you know how to use QR codes?)	Yes	No
3. Would you be willing to receive a validated patient information leaflet using QR codes?	Yes	No
Section 3 (To be completed by clinician after clinic appointment)		
1. Was a validated patient information leaflet provided?	Yes	No
If yes, please provide details below		

**Table 5:** Second phase questionnaire.

Clinic date:		
Age:		
Gender:		
To what degree do you agree or disagree with the following statements? (Please circle your answer)		
No.	Statement	Response options
1	“It was easy to scan the QR code.”	Strongly agree/Agree/Neutral/Disagree/Strongly disagree
2	“The QR code was convenient to use.”	Strongly agree/Agree/Neutral/Disagree/Strongly disagree
3	“I would prefer to use a QR code to access the validated patient information leaflet instead of a physical copy.”	Strongly agree/Agree/Neutral/Disagree/Strongly disagree
Additional comments:		
